# Supplementary material for: Diagnostic potential for a serum miRNA neural network for detection of ovarian cancer
Source: eLife. 2017 Oct 31;6:e28932. doi: 10.7554/eLife.28932 (PMC5679755; doi:10.7554/eLife.28932)
Supplement: Supplementary file 1. — (A) Area under the ROC curve analyses for the various testing methods depending on the variable selection protocol using data without batch adjustment. Like the batch-adjusted data, the neural network using the fold change variable outperformed the other methods in terms of classifier accuracy and did not overfit the predictions to the training set. (B) Individual sample predictions of the tested classification models built on the unadjusted fold change-based variable selection miRNA subset. [file elife-28932-supp1.docx]

**Supplementary File 1A. Area under the ROC curve analyses for the various testing methods depending on the variable selection protocol using data without batch adjustment.** Similar to the batch-adjusted data, the neural network using the fold change variable outperformed the other methods in terms of classifier accuracy and did not overfit the predictions to the training set.

|  | Training set (N=135) | | | Testing set (N=44) | | |
| --- | --- | --- | --- | --- | --- | --- |
|  | Variable selection | | | Variable selection | | |
| Method | Significance-based | CFS subset | Fold change | Significance-based | CFS subset | Fold change |
| LDA | 0.81 (0.74-0.88) | 0.76 (0.68-0.84) | 0.73 (0.65-0.82) | 0.78 (0.65-0.92) | 0.75 (0.60-0.89) | 0.74 (0.59-0.88) |
| LR | 0.82 (0.75-0.89) | 0.76 (0.68-0.84) | 0.74 (0.65-0.82) | 0.81 (0.68-0.94) | 0.75 (0.60-0.89) | 0.78 (0.64-0.92) |
| NN | 0.89 (0.84-0.95) | 0.80 (0.73-0.88) | 0.93 (0.89-0.98) | 0.77 (0.63-0.91) | 0.71 (0.56-0.86) | 0.90 (0.80-0.99) |
| SVM | 0.82 (0.75-0.89) | 0.66 (0.57-0.75) | 0.87 (0.81-0.93) | 0.74 (0.60-0.89) | 0.66 (0.50-0.82) | 0.74 (0.60-0.89) |
| MARS | 0.87 (0.81-0.93) | 0.84 (0.77-0.91) | 0.79 (0.71-0.86) | 0.56 (0.39-0.73) | 0.69 (0.54-0.85) | 0.67 (0.51-0.83) |
| Bayes | 0.81 (0.73-0.88) | 0.80 (0.73-0.87) | 0.80 (0.73-0.87) | 0.74 (0.60-0.89) | 0.64 (0.48-0.81) | 0.72 (0.57-0.87) |
| LADtree | 0.97 (0.94-1.00) | 0.96 (0.92-0.99) | 0.96 (0.92-0.99) | 0.64 (0.48-0.81) | 0.67 (0.51-0.83) | 0.59 (0.42-0.76) |
| FT | 0.81 (0.74-0.89) | 0.75 (0.66-0.83) | 0.83 (0.77-0.90) | 0.81 (0.68-0.94) | 0.60 (0.44-0.77) | 0.82 (0.70-0.95) |
| Bayes  Net | 0.80 (0.73-0.88) | 0.80 (0.73-0.88) | 0.80 (0.73-0.88) | 0.65 (0.49-0.81) | 0.65 (0.49-0.81) | 0.65 (0.49-0.81) |
| RF | 1.00 (1.00-1.00) | 1.00 (1.00-1.00) | 1.00 (1.00-1.00) | 0.77 (0.63-0.91) | 0.72 (0.57-0.87) | 0.68 (0.52-0.84) |
| EN | 0.83 (0.76-0.90) | 0.78 (0.70-0.85) | 0.81 (0.74-0.88) | 0.80 (0.67-0.93) | 0.72 (0.57-0.87) | 0.80 (0.66-0.93) |
| LDA - Linear discriminant analysis  LG - Logistic regression  NN – Neural network  SVM - Support vector machine  Bayes – Naive Bayes  MARS - Multivariate adaptive regression splines  FT – Functional tree  Bayes net - Bayesian network  RF – Random forest  EN – Elastic network regression  95% Confidence intervals in parentheses | | | | | | |

**Supplementary File 1B Individual sample predictions of the tested classification models built on the unadjusted fold change-based variable selection miRNA subset.**

| ID | Study | Stage | Grade | Histology | Age | CA-125 | LDA | LR | NN | SVM | Bayes | MARS | LAD Tree | FT | Bayes net | RF | EN | CA-125 >35 |
| --- | --- | --- | --- | --- | --- | --- | --- | --- | --- | --- | --- | --- | --- | --- | --- | --- | --- | --- |
| 1 | NECC | * | * | control | 53 | ‡ | BBC | CA | BBC | BBC | CA | BBC | BBC | BBC | CA | BBC | BBC | * |
| 2 | PMP | * | 0 | serous borderline | 58 | 763 | CA | CA | BBC | CA | CA | BBC | BBC | CA | CA | BBC | CA | CA |
| 3 | PMP | III | 3 | serous adenocarcinoma | 59 | 25.8 | BBC | CA | BBC | CA | CA | BBC | CA | BBC | BBC | CA | BBC | BBC |
| 4 | PMP | IV | 3 | serous adenocarcinoma | 66 | ‡ | CA | CA | CA | CA | BBC | CA | CA | CA | CA | CA | CA | * |
| 5 | PMP | * | * | endometrioma | 49 | ‡ | BBC | BBC | BBC | BBC | BBC | BBC | BBC | BBC | BBC | BBC | BBC | * |
| 6 | PMP | I | 1 | clear cell adenocarcinoma | 50 | 13 | BBC | CA | CA | CA | BBC | BBC | CA | BBC | CA | CA | BBC | BBC |
| 7 | PMP | * | * | serous cystadenoma | 47 | ‡ | BBC | BBC | BBC | BBC | BBC | BBC | BBC | BBC | BBC | BBC | CA | * |
| 8 | PMP | I | 2 | endometrioid adenocarcinoma | 49 | ‡ | CA | CA | CA | CA | CA | CA | CA | CA | CA | CA | CA | * |
| 9 | PMP | III | 3 | clear cell adenocarcinoma | 54 | 87.6 | BBC | BBC | CA | CA | CA | CA | BBC | BBC | BBC | CA | BBC | CA |
| 10 | PMP | * | * | serous cystadenoma | 45 | 17.4 | BBC | CA | BBC | BBC | BBC | BBC | BBC | BBC | BBC | BBC | CA | BBC |
| 11 | PMP | * | 0 | serous borderline | 59 | 42.8 | BBC | BBC | BBC | BBC | BBC | BBC | BBC | BBC | BBC | BBC | BBC | CA |
| 12 | PMP | II | 2 | clear cell adenocarcinoma | 59 | 125 | CA | CA | CA | CA | CA | CA | CA | CA | CA | CA | CA | CA |
| 13 | PMP | * | * | serous cystadenoma | 70 | 12 | BBC | BBC | CA | BBC | BBC | BBC | BBC | BBC | BBC | BBC | BBC | BBC |
| 14 | PMP | III | 3 | endometrioid adenocarcinoma | 57 | 382.2 | BBC | BBC | CA | CA | CA | CA | CA | BBC | BBC | CA | BBC | CA |
| 15 | PMP | * | 0 | serous borderline | 56 | 450.3 | BBC | CA | CA | CA | CA | BBC | BBC | CA | CA | BBC | CA | CA |
| 16 | PMP | III | 3 | endometrioid adenocarcinoma | 63 | 1002 | CA | CA | CA | CA | CA | CA | CA | CA | CA | CA | CA | CA |
| 17 | PMP | * | 0 | serous borderline | 69 | 388 | BBC | BBC | BBC | CA | CA | CA | CA | BBC | BBC | CA | BBC | CA |
| 18 | PMP | * | 0 | serous borderline | 50 | 118 | BBC | BBC | BBC | BBC | BBC | BBC | BBC | BBC | BBC | BBC | CA | CA |
| 19 | PMP | III | 2 | endometrioid adenocarcinoma | 55 | ‡ | CA | CA | CA | CA | CA | CA | CA | CA | CA | CA | CA | * |
| 20 | PMP | II | 3 | serous adenocarcinoma | 74 | 9 | CA | CA | BBC | CA | CA | CA | CA | CA | BBC | CA | CA | BBC |
| 21 | PMP | * | * | endometrioma | 65 | 48 | CA | CA | BBC | BBC | CA | CA | CA | BBC | BBC | BBC | CA | CA |
| 22 | PMP | * | * | endometrioma | 52 | ‡ | BBC | BBC | CA | BBC | BBC | BBC | CA | BBC | BBC | BBC | BBC | * |
| 23 | PMP | * | 0 | serous borderline | 51 | ‡ | BBC | BBC | BBC | BBC | BBC | BBC | BBC | BBC | BBC | BBC | BBC | * |
| 24 | PMP | I | 1 | clear cell adenocarcinoma | 64 | 30.5 | CA | CA | CA | CA | CA | CA | CA | CA | CA | CA | CA | BBC |
| 25 | PMP | * | 0 | serous borderline | 56 | 9.4 | BBC | BBC | CA | BBC | BBC | BBC | BBC | BBC | BBC | BBC | BBC | BBC |
| 26 | PMP | III | 3 | serous adenocarcinoma | 55 | 277.2 | CA | CA | CA | CA | CA | CA | CA | CA | CA | CA | CA | CA |
| 27 | PMP | * | * | endometrioma | 55 | ‡ | BBC | BBC | BBC | BBC | BBC | BBC | BBC | BBC | BBC | BBC | BBC | * |
| 28 | NECC | * | * | control | 45 | ‡ | BBC | BBC | BBC | BBC | BBC | BBC | CA | CA | CA | CA | BBC | * |
| 29 | NECC | * | * | control | 71 | ‡ | BBC | BBC | BBC | BBC | BBC | BBC | BBC | BBC | BBC | BBC | BBC | * |
| 30 | PMP | * | * | endometrioma | 60 | 10.5 | CA | CA | BBC | BBC | BBC | BBC | BBC | CA | BBC | BBC | CA | BBC |
| 31 | PMP | * | * | serous cystadenoma | 59 | ‡ | BBC | BBC | BBC | BBC | BBC | BBC | BBC | BBC | BBC | BBC | BBC | * |
| 32 | PMP | II | 3 | serous adenocarcinoma | 63 | 568 | CA | CA | CA | CA | CA | CA | CA | CA | CA | CA | CA | CA |
| 33 | PMP | III | 3 | serous adenocarcinoma | 48 | 3723 | CA | CA | CA | CA | CA | CA | CA | CA | CA | CA | CA | CA |
| 34 | NECC | * | * | control | 58 | ‡ | BBC | BBC | BBC | BBC | BBC | BBC | BBC | BBC | BBC | BBC | BBC | * |
| 35 | PMP | * | * | serous cystadenoma | 55 | ‡ | CA | BBC | BBC | BBC | CA | CA | BBC | BBC | BBC | CA | CA | * |
| 36 | PMP | II | 3 | serous adenocarcinoma | 52 | 49 | CA | CA | CA | CA | CA | CA | CA | CA | CA | CA | CA | CA |
| 37 | PMP | II | 3 | serous adenocarcinoma | 59 | 7000 | CA | CA | CA | CA | BBC | CA | CA | CA | CA | CA | CA | CA |
| 38 | NECC | * | * | control | 60 | ‡ | BBC | BBC | BBC | BBC | BBC | BBC | BBC | BBC | BBC | BBC | BBC | * |
| 39 | PMP | III | 3 | serous adenocarcinoma | 49 | ‡ | BBC | CA | CA | CA | BBC | CA | CA | CA | CA | CA | CA | * |
| 40 | PMP | * | * | endometrioma | 56 | 70 | BBC | BBC | BBC | BBC | BBC | BBC | BBC | BBC | BBC | BBC | BBC | CA |
| 41 | PMP | II | 2 | endometrioid adenocarcinoma | 52 | 1889 | CA | CA | CA | CA | CA | CA | CA | CA | CA | CA | CA | CA |
| 42 | NECC | * | * | control | 59 | ‡ | BBC | BBC | BBC | BBC | BBC | BBC | BBC | BBC | BBC | BBC | BBC | * |
| 43 | PMP | II | 3 | serous adenocarcinoma | 69 | 213 | CA | CA | CA | CA | BBC | CA | CA | CA | CA | CA | CA | CA |
| 44 | PMP | * | * | endometrioma | 50 | 5 | BBC | BBC | BBC | BBC | BBC | BBC | BBC | BBC | BBC | BBC | BBC | BBC |
| 45 | PMP | I | 1 | endometrioid adenocarcinoma | 57 | 23 | CA | CA | CA | CA | BBC | CA | CA | CA | CA | CA | CA | BBC |
| 46 | NECC | * | * | control | 52 | ‡ | BBC | BBC | BBC | BBC | BBC | BBC | BBC | BBC | BBC | BBC | BBC | * |
| 47 | PMP | * | * | endometrioma | 53 | 336 | BBC | CA | BBC | BBC | BBC | CA | BBC | BBC | BBC | BBC | BBC | CA |
| 48 | PMP | II | 3 | serous adenocarcinoma | 58 | 268 | CA | CA | CA | CA | CA | CA | CA | CA | CA | CA | CA | CA |
| 49 | PMP | * | * | endometrioma | 56 | ‡ | BBC | BBC | BBC | BBC | BBC | BBC | CA | BBC | BBC | BBC | BBC | * |
| 50 | PMP | I | 2 | endometrioid adenocarcinoma | 66 | 31.2 | BBC | BBC | BBC | BBC | BBC | CA | BBC | CA | BBC | CA | BBC | BBC |
| 51 | PMP | * | 1 | endometrioid adenocarcinoma | 62 | 1726 | CA | CA | CA | CA | CA | CA | CA | CA | CA | CA | CA | CA |
| 52 | NECC | * | * | control | 63 | ‡ | BBC | BBC | BBC | BBC | BBC | BBC | BBC | BBC | BBC | BBC | BBC | * |
| 53 | PMP | * | 0 | serous borderline | 54 | 4.9 | BBC | BBC | BBC | BBC | BBC | BBC | CA | BBC | CA | CA | BBC | BBC |
| 54 | PMP | II | 3 | serous adenocarcinoma | 59 | 107.9 | CA | CA | CA | CA | BBC | CA | CA | CA | CA | CA | CA | CA |
| 55 | PMP | II | 3 | serous adenocarcinoma | 60 | 23.1 | BBC | BBC | BBC | BBC | BBC | BBC | BBC | BBC | BBC | CA | BBC | BBC |
| 57 | PMP | I | 2 | endometrioid adenocarcinoma | 59 | ‡ | BBC | BBC | CA | BBC | BBC | BBC | BBC | BBC | BBC | BBC | BBC | * |
| 58 | PMP | * | 0 | serous borderline | 69 | ‡ | CA | CA | BBC | BBC | BBC | BBC | BBC | BBC | BBC | BBC | BBC | * |
| 59 | PMP | I | 3 | serous adenocarcinoma | 61 | 302.6 | CA | CA | CA | CA | CA | CA | CA | CA | CA | CA | CA | CA |
| 60 | PMP | III | 3 | endometrioid adenocarcinoma | 56 | 58 | CA | CA | CA | CA | BBC | CA | CA | CA | CA | CA | CA | CA |
| 61 | PMP | * | * | endometrioma | 55 | 172.4 | BBC | BBC | BBC | BBC | BBC | BBC | BBC | BBC | BBC | BBC | BBC | CA |
| 62 | PMP | I | 2 | endometrioid adenocarcinoma | 68 | ‡ | CA | CA | CA | CA | BBC | CA | CA | BBC | CA | CA | BBC | * |
| 63 | PMP | II | 3 | serous adenocarcinoma | 52 | 367.3 | CA | CA | CA | CA | CA | CA | CA | CA | CA | CA | CA | CA |
| 64 | PMP | * | 0 | serous borderline | 45 | 16.5 | BBC | BBC | BBC | BBC | BBC | BBC | BBC | BBC | BBC | BBC | BBC | BBC |
| 65 | PMP | I | 1 | serous adenocarcinoma | 60 | ‡ | BBC | BBC | CA | CA | BBC | BBC | CA | BBC | BBC | CA | BBC | * |
| 66 | PMP | III | 3 | serous adenocarcinoma | 51 | ‡ | CA | CA | CA | CA | CA | CA | CA | CA | CA | CA | CA | * |
| 67 | PMP | I | 3 | serous adenocarcinoma | 56 | ‡ | BBC | BBC | BBC | BBC | BBC | BBC | CA | BBC | BBC | CA | BBC | * |
| 68 | PMP | * | 0 | serous borderline | 49 | ‡ | BBC | BBC | BBC | BBC | BBC | BBC | BBC | BBC | BBC | BBC | BBC | * |
| 69 | PMP | III | 3 | serous adenocarcinoma | 70 | 1212 | CA | CA | CA | CA | CA | CA | CA | CA | CA | CA | CA | CA |
| 70 | PMP | I | 1 | clear cell adenocarcinoma | 51 | ‡ | CA | CA | CA | CA | BBC | BBC | CA | CA | CA | CA | CA | * |
| 71 | PMP | I | 2 | endometrioid adenocarcinoma | 57 | 209.2 | CA | CA | CA | CA | CA | CA | CA | BBC | BBC | CA | BBC | CA |
| 72 | PMP | * | 0 | serous borderline | 52 | 42 | BBC | BBC | BBC | BBC | BBC | BBC | BBC | BBC | BBC | BBC | BBC | CA |
| 73 | PMP | I | 3 | serous adenocarcinoma | 52 | 38.9 | CA | CA | CA | CA | CA | CA | CA | CA | BBC | CA | CA | CA |
| 74 | PMP | I | 1 | endometrioid adenocarcinoma | 71 | ‡ | BBC | BBC | BBC | BBC | BBC | BBC | CA | BBC | BBC | BBC | BBC | * |
| 75 | PMP | * | * | serous cystadenoma | 56 | ‡ | BBC | CA | BBC | BBC | BBC | CA | BBC | BBC | BBC | BBC | BBC | * |
| 76 | NECC | * | * | control | 45 | ‡ | BBC | BBC | BBC | BBC | CA | BBC | BBC | BBC | BBC | BBC | CA | * |
| 77 | PMP | * | * | serous cystadenoma | 69 | ‡ | BBC | BBC | CA | BBC | BBC | BBC | CA | BBC | BBC | BBC | BBC | * |
| 78 | NECC | * | * | control | 47 | ‡ | BBC | BBC | BBC | BBC | BBC | BBC | CA | BBC | CA | CA | BBC | * |
| 79 | PMP | * | * | serous cystadenoma | 51 | 87.2 | CA | CA | BBC | CA | BBC | BBC | BBC | BBC | BBC | BBC | CA | CA |
| 80 | PMP | III | 3 | serous adenocarcinoma | 59 | ‡ | CA | CA | CA | CA | CA | CA | CA | CA | CA | CA | CA | * |
| 81 | PMP | * | * | endometrioma | 54 | 20.4 | BBC | BBC | BBC | BBC | CA | CA | CA | BBC | BBC | CA | BBC | BBC |
| 82 | PMP | * | 0 | serous borderline | 60 | 6.2 | BBC | BBC | BBC | BBC | BBC | BBC | BBC | BBC | BBC | BBC | BBC | BBC |
| 83 | NECC | * | * | control | 49 | ‡ | BBC | BBC | BBC | BBC | BBC | BBC | BBC | BBC | BBC | BBC | BBC | * |
| 84 | NECC | * | * | control | 48 | ‡ | BBC | BBC | CA | BBC | BBC | BBC | BBC | BBC | BBC | BBC | BBC | * |
| 85 | PMP | * | * | serous cystadenoma | 55 | ‡ | CA | CA | CA | CA | CA | CA | CA | CA | CA | CA | CA | * |
| 86 | PMP | I | 3 | serous adenocarcinoma | 53 | 124 | BBC | BBC | CA | BBC | BBC | BBC | CA | BBC | BBC | BBC | BBC | CA |
| 87 | PMP | * | * | serous cystadenoma | 71 | ‡ | CA | CA | BBC | CA | CA | BBC | CA | BBC | CA | BBC | CA | * |
| 88 | PMP | I | 1 | endometrioid adenocarcinoma | 65 | 25 | BBC | BBC | BBC | BBC | BBC | BBC | CA | BBC | BBC | CA | CA | BBC |
| 89 | PMP | * | * | endometrioma | 52 | ‡ | CA | CA | BBC | BBC | CA | BBC | BBC | BBC | CA | BBC | CA | * |
| 90 | PMP | * | * | endometrioma | 56 | ‡ | BBC | BBC | BBC | BBC | BBC | BBC | BBC | BBC | BBC | BBC | BBC | * |
| 91 | NECC | * | * | control | 52 | ‡ | CA | CA | CA | CA | CA | BBC | BBC | CA | CA | BBC | CA | * |
| 92 | PMP | III | 3 | serous adenocarcinoma | 50 | 7005 | CA | CA | CA | CA | CA | CA | CA | CA | CA | CA | CA | CA |
| 93 | PMP | I | 1 | endometrioid adenocarcinoma | 47 | 25.6 | BBC | BBC | CA | CA | BBC | BBC | CA | CA | CA | CA | CA | BBC |
| 94 | PMP | II | 3 | endometrioid adenocarcinoma | 56 | 129 | CA | CA | CA | CA | BBC | CA | CA | CA | CA | CA | CA | CA |
| 95 | PMP | III | 3 | serous adenocarcinoma | 60 | ‡ | CA | CA | CA | CA | CA | CA | CA | CA | CA | CA | CA | * |
| 96 | PMP | * | * | endometrioma | 70 | ‡ | BBC | BBC | BBC | BBC | BBC | BBC | BBC | BBC | BBC | BBC | BBC | * |
| 97 | PMP | III | 2 | endometrioid adenocarcinoma | 51 | 3257 | CA | CA | CA | CA | CA | CA | CA | CA | BBC | CA | CA | CA |
| 98 | NECC | * | * | control | 67 | ‡ | BBC | BBC | BBC | BBC | BBC | BBC | CA | BBC | BBC | BBC | BBC | * |
| 99 | PMP | I | 3 | endometrioid adenocarcinoma | 56 | 35.2 | CA | CA | CA | CA | CA | CA | CA | CA | CA | CA | CA | CA |
| 100 | PMP | II | 3 | serous adenocarcinoma | 52 | 1132 | CA | CA | CA | CA | CA | CA | CA | CA | CA | CA | CA | CA |
| 101 | PMP | II | 3 | mixed with clear cell adenocarcinoma or endometroid component | 59 | 542.6 | CA | CA | CA | CA | CA | CA | CA | CA | CA | CA | CA | CA |
| 102 | PMP | * | * | serous cystadenoma | 53 | ‡ | BBC | BBC | BBC | BBC | CA | BBC | BBC | BBC | BBC | BBC | BBC | * |
| 103 | NECC | * | * | control | 53 | ‡ | BBC | BBC | BBC | BBC | BBC | BBC | BBC | BBC | BBC | BBC | CA | * |
| 104 | PMP | III | 2 | clear cell adenocarcinoma | 62 | ‡ | CA | CA | CA | CA | CA | CA | CA | CA | CA | CA | CA | * |
| 105 | PMP | I | 1 | endometrioid adenocarcinoma | 45 | 31.4 | BBC | BBC | BBC | CA | BBC | CA | CA | CA | CA | CA | BBC | BBC |
| 106 | PMP | II | 3 | serous adenocarcinoma | 48 | 681 | CA | CA | CA | CA | CA | CA | CA | CA | CA | CA | CA | CA |
| 107 | PMP | II | 2 | serous adenocarcinoma | 55 | 88.6 | CA | CA | CA | CA | CA | CA | CA | CA | CA | CA | CA | CA |
| 108 | PMP | III | 3 | clear cell adenocarcinoma | 50 | 721.1 | CA | CA | CA | CA | CA | CA | CA | CA | CA | CA | CA | CA |
| 109 | PMP | * | * | serous cystadenoma | 50 | ‡ | BBC | BBC | BBC | BBC | BBC | BBC | BBC | BBC | BBC | BBC | CA | * |
| 110 | PMP | * | * | serous cystadenoma | 52 | ‡ | BBC | BBC | BBC | BBC | BBC | BBC | BBC | CA | BBC | BBC | CA | * |
| 111 | PMP | * | * | serous cystadenoma | 49 | ‡ | BBC | BBC | BBC | BBC | CA | BBC | BBC | BBC | BBC | BBC | BBC | * |
| 112 | PMP | III | 3 | mixed with clear cell adenocarcinoma or endometroid component | 57 | 20.9 | CA | CA | CA | CA | CA | CA | CA | CA | CA | CA | CA | BBC |
| 113 | PMP | I | 1 | endometrioid adenocarcinoma | 65 | ‡ | BBC | BBC | CA | CA | CA | BBC | CA | CA | CA | CA | CA | * |
| 114 | PMP | II | 3 | serous adenocarcinoma | 45 | 19.4 | CA | CA | CA | CA | CA | CA | BBC | CA | BBC | BBC | CA | BBC |
| 115 | PMP | I | 2 | serous adenocarcinoma | 52 | ‡ | CA | CA | CA | CA | BBC | BBC | CA | CA | BBC | CA | CA | * |
| 116 | PMP | I | 1 | serous adenocarcinoma | 73 | 128 | BBC | CA | BBC | BBC | BBC | BBC | CA | BBC | CA | CA | BBC | CA |
| 117 | PMP | * | 0 | serous borderline | 62 | 42 | BBC | BBC | BBC | BBC | BBC | BBC | BBC | BBC | CA | BBC | BBC | CA |
| 118 | PMP | * | 0 | serous borderline | 61 | ‡ | BBC | BBC | BBC | BBC | BBC | BBC | BBC | BBC | BBC | BBC | BBC | * |
| 119 | PMP | II | 3 | endometrioid adenocarcinoma | 46 | 371 | CA | CA | CA | CA | CA | CA | CA | CA | CA | CA | CA | CA |
| 120 | PMP | * | * | endometrioma | 55 | 4.2 | BBC | BBC | BBC | CA | CA | CA | CA | CA | CA | CA | CA | BBC |
| e1001 | ERA | II | 2 | endometrioid adenocarcinoma | 59 | ‡ | CA | CA | CA | CA | CA | CA | CA | CA | CA | CA | CA | * |
| e1002 | ERA | III | 3 | serous adenocarcinoma | 70 | 2589 | BBC | BBC | CA | CA | BBC | CA | CA | CA | BBC | CA | BBC | CA |
| e1003 | ERA | II | 1 | endometrioid adenocarcinoma | 70 | 484 | CA | CA | CA | CA | CA | CA | CA | CA | CA | CA | CA | CA |
| e1004 | ERA | III | 3 | serous adenocarcinoma | 59 | 3011 | CA | CA | CA | CA | CA | CA | CA | CA | CA | CA | CA | CA |
| e1005 | ERA | II | 3 | serous adenocarcinoma | 56 | 302.6 | CA | CA | CA | CA | CA | CA | BBC | CA | CA | CA | CA | CA |
| e1006 | ERA | * | * | fibrothecoma | 52 | 45 | BBC | CA | CA | CA | CA | CA | CA | BBC | CA | BBC | CA | CA |
| e1007 | ERA | III | 3 | serous adenocarcinoma | 75 | 132 | CA | CA | CA | CA | CA | CA | CA | CA | CA | CA | CA | CA |
| e1008 | ERA | I | 3 | serous adenocarcinoma | 66 | 135 | CA | CA | CA | CA | CA | CA | CA | CA | BBC | CA | CA | CA |
| e1009 | ERA | IV | 3 | serous adenocarcinoma | 55 | 2974.8 | CA | CA | CA | CA | CA | CA | CA | CA | CA | CA | CA | CA |
| e1010 | ERA | * | * | simple Cyst | 51 | 795.6 | CA | CA | BBC | BBC | BBC | CA | BBC | BBC | BBC | BBC | BBC | CA |
| e1011 | ERA | III | 3 | serous adenocarcinoma | 66 | 152.7 | BBC | CA | CA | CA | CA | CA | CA | CA | CA | CA | CA | CA |
| e1012 | ERA | III | 0 | serous borderline | 61 | 274 | CA | CA | BBC | CA | CA | CA | CA | CA | CA | CA | CA | CA |
| e1013 | ERA | I | 0 | mucinous borderline | 48 | 10.7 | BBC | BBC | BBC | BBC | BBC | BBC | BBC | BBC | BBC | BBC | BBC | BBC |
| e1014 | ERA | IV | 3 | serous adenocarcinoma | 59 | 252.3 | CA | CA | CA | CA | CA | CA | CA | CA | BBC | CA | CA | CA |
| e1015 | ERA | III | 3 | serous adenocarcinoma | 59 | 300 | CA | CA | CA | CA | CA | CA | CA | CA | CA | CA | CA | CA |
| e1016 | ERA | II | 3 | clear cell adenocarcinoma | 51 | ‡ | CA | CA | CA | CA | CA | CA | CA | CA | CA | CA | CA | * |
| e1017 | ERA | II | 3 | serous adenocarcinoma | 61 | 32.6 | CA | CA | CA | CA | CA | CA | CA | CA | CA | CA | CA | BBC |
| e1019 | ERA | III | 3 | serous adenocarcinoma | 71 | ‡ | CA | CA | CA | CA | CA | CA | CA | CA | BBC | CA | CA | * |
| e1020 | ERA | III | 3 | serous adenocarcinoma | 73 | ‡ | BBC | BBC | CA | BBC | BBC | CA | BBC | BBC | BBC | BBC | BBC | * |
| e1021 | ERA | IV | 3 | serous adenocarcinoma | 49 | 225.9 | CA | CA | CA | CA | BBC | BBC | CA | BBC | BBC | CA | CA | CA |
| e1022 | ERA | II | 3 | serous adenocarcinoma | 65 | 442 | CA | CA | CA | CA | CA | CA | CA | CA | CA | CA | CA | CA |
| e1023 | ERA | I | 1 | endometrioid adenocarcinoma | 65 | 416.2 | BBC | BBC | BBC | BBC | BBC | BBC | BBC | BBC | BBC | CA | BBC | CA |
| e1024 | ERA | III | 1 | serous adenocarcinoma | 46 | 464 | BBC | BBC | CA | CA | CA | CA | CA | BBC | BBC | CA | BBC | CA |
| e1026 | ERA | III | 3 | metastatic uterine myxoid leiomyosarcoma | 48 | 54.2 | CA | CA | CA | CA | CA | CA | CA | CA | CA | CA | CA | CA |
| e1027 | ERA | III | 0 | serous borderline | 45 | 377 | BBC | BBC | BBC | BBC | BBC | BBC | BBC | BBC | BBC | BBC | BBC | CA |
| e1029 | ERA | * | * | adenofibroma | 28 | 12.3 | BBC | BBC | BBC | BBC | BBC | BBC | CA | BBC | BBC | BBC | BBC | BBC |
| e1030 | ERA | IV | 3 | metastatic uterine papillary serous carcinoma | 68 | 482.9 | CA | CA | CA | CA | CA | CA | CA | CA | CA | CA | CA | CA |
| e1031 | ERA | III | 3 | serous adenocarcinoma | 64 | 278 | CA | BBC | CA | CA | BBC | BBC | BBC | BBC | BBC | BBC | CA | CA |
| e1032 | ERA | III | 3 | serous adenocarcinoma | 56 | 1369 | CA | CA | CA | CA | CA | CA | CA | CA | CA | CA | CA | CA |
| e1033 | ERA | IV | 3 | metastatic uterine epithelioid leiomyosarcoma | 66 | 16.8 | CA | CA | BBC | CA | CA | CA | CA | CA | BBC | CA | CA | BBC |
| e1034 | ERA | IV | 2 | metastatic endometrioid endometrial adenocarcinoma | 49 | 30.5 | BBC | BBC | CA | BBC | BBC | BBC | CA | BBC | BBC | CA | BBC | BBC |
| e1035 | ERA | II | 3 | serous adenocarcinoma | 59 | 40.4 | BBC | BBC | CA | CA | BBC | CA | CA | CA | BBC | CA | BBC | CA |
| e1036 | ERA | * | * | serous cystadenoma | 50 | 7.5 | BBC | BBC | BBC | BBC | BBC | BBC | BBC | BBC | BBC | BBC | BBC | BBC |
| e1037 | ERA | I | 0 | mucinous borderline | 54 | 49.8 | BBC | BBC | BBC | BBC | BBC | BBC | BBC | BBC | BBC | BBC | CA | CA |
| e1038 | ERA | * | * | serous cystadenofibroma | 72 | 21 | CA | CA | BBC | BBC | CA | CA | CA | BBC | CA | CA | CA | BBC |
| e1039 | ERA | * | * | serous cystadenoma | 55 | ‡ | BBC | BBC | BBC | BBC | BBC | BBC | BBC | BBC | CA | BBC | BBC | * |
| e1040 | ERA | * | * | benign Brenner tumor | 44 | 6 | BBC | BBC | CA | BBC | BBC | BBC | BBC | CA | BBC | BBC | BBC | BBC |
| e1041 | ERA | III | 3 | serous adenocarcinoma | 78 | 92 | CA | CA | CA | CA | CA | CA | CA | CA | CA | CA | CA | CA |
| e1042 | ERA | * | * | serous cystadenofibroma | 52 | 16.1 | BBC | BBC | BBC | BBC | BBC | BBC | BBC | BBC | BBC | BBC | BBC | BBC |
| e1043 | ERA | * | * | serous cystadenofibroma | 55 | 87.2 | BBC | BBC | BBC | BBC | BBC | BBC | CA | BBC | BBC | BBC | BBC | CA |
| e1044 | ERA | III | 3 | serous adenocarcinoma | 62 | 200.5 | CA | CA | CA | CA | CA | CA | CA | CA | CA | CA | CA | CA |
| e1045 | ERA | III | 3 | serous adenocarcinoma | 69 | 326 | CA | CA | CA | CA | BBC | CA | CA | CA | CA | CA | CA | CA |
| e1046 | ERA | I | 0 | serous borderline | 53 | 172.7 | BBC | BBC | BBC | BBC | BBC | BBC | CA | BBC | BBC | BBC | BBC | CA |
| e1047 | ERA | III | 3 | serous adenocarcinoma | 68 | ‡ | CA | BBC | CA | CA | CA | CA | CA | CA | CA | CA | CA | * |
| e1048 | ERA | III | 0 | serous borderline | 53 | 133 | BBC | BBC | CA | CA | BBC | CA | BBC | CA | BBC | CA | BBC | CA |
| e1049 | ERA | II | 2 | endometrioid adenocarcinoma | 52 | 1889 | CA | CA | CA | CA | CA | CA | CA | CA | CA | CA | CA | CA |
| e1050 | ERA | I | 0 | serous borderline | 56 | 7.3 | CA | CA | CA | CA | CA | CA | CA | CA | CA | CA | CA | BBC |
| e1051 | ERA | IV | 1 | serous adenocarcinoma | 43 | 53 | BBC | BBC | BBC | BBC | CA | CA | CA | CA | CA | CA | CA | CA |
| e1052 | ERA | I | 3 | endometrioid adenocarcinoma | 56 | 157.3 | CA | CA | CA | CA | CA | CA | CA | CA | CA | CA | CA | CA |
| e1053 | ERA | * | * | atypical leiomyoma | 44 | 114.2 | CA | BBC | CA | CA | BBC | CA | BBC | BBC | BBC | BBC | BBC | CA |
| e1054 | ERA | * | * | mucinous cystadenoma | 63 | 26 | BBC | BBC | BBC | BBC | BBC | BBC | BBC | BBC | BBC | BBC | BBC | BBC |
| e1055 | ERA | * | * | serous cystadenofibroma | 61 | 381 | BBC | BBC | BBC | BBC | BBC | BBC | BBC | BBC | BBC | BBC | BBC | CA |
| e1056 | ERA | I | 1 | mucinous adenocarcinoma | 50 | 228 | BBC | BBC | CA | CA | BBC | CA | CA | CA | BBC | CA | BBC | CA |
| e1057 | ERA | III | 3 | metastatic endometrioid endometrial adenocarcinoma | 58 | 257 | BBC | BBC | BBC | CA | CA | CA | CA | CA | CA | CA | BBC | CA |
| e1058 | ERA | * | * | benign Brenner tumor | 63 | 23 | BBC | BBC | BBC | BBC | BBC | BBC | BBC | BBC | BBC | BBC | BBC | BBC |
| e1059 | ERA | I | 1 | adult Granulosa Cell tumor | 50 | 15.3 | BBC | BBC | CA | BBC | BBC | CA | CA | BBC | BBC | CA | BBC | BBC |
| e1060 | ERA | * | * | fibroma | 60 | 27 | BBC | BBC | BBC | BBC | BBC | BBC | BBC | BBC | BBC | BBC | BBC | BBC |
| e1061 | ERA | IV | 3 | serous adenocarcinoma | 52 | 536 | CA | CA | CA | CA | CA | CA | CA | CA | CA | CA | CA | CA |
| e1062 | ERA | * | * | serous cystadenoma | 77 | 99 | BBC | BBC | BBC | BBC | BBC | BBC | CA | BBC | BBC | BBC | BBC | CA |
| e1063 | ERA | * | * | leiomyoma | 43 | 228 | CA | CA | CA | CA | BBC | BBC | BBC | CA | BBC | CA | CA | CA |
| PMP – Pelvic Mass Protocol  NECC – New England Case Control Study  ERA – Effects of Regional Analgesia on Serum miRNA after Oncology Surgery Study  * - Not applicable; ‡ Not available  LDA - Linear discriminant analysis  LR - Logistic regression  NN – Neural network  SVM - Support vector machine  Bayes – Naive Bayes  MARS - Multivariate adaptive regression splines  FT – Functional tree  Bayes net - Bayesian network  RF – Random forest  EN – Elastic network regression  CA-125>35 – Serum CA-125 level greater than 35 unit/ml  BBC – Benign, Borderline, or Control  CA - Cancer | | | | | | | | | | | | | | | | | | |
