# Supplementary material for: Diagnostic potential for a serum miRNA neural network for detection of ovarian cancer
Source: eLife. 2017 Oct 31;6:e28932. doi: 10.7554/eLife.28932 (PMC5679755; doi:10.7554/eLife.28932)
Supplement: Supplementary file 2. — (A) Misclassification matrices for the neural network and CA125 predictions with detailed histopathological data. (B) Misclassification matrices for the neural network stratified by age. (C) miRNA expression by tumor histology and stage. [file elife-28932-supp2.docx]

**Supplementary File 2A. Misclassification matrices for the neural network and CA125 predictions with detailed histopathological data.**

|  | **CA-125 Prediction** | | | **Neural Network Prediction** | | | **p-value** |
| --- | --- | --- | --- | --- | --- | --- | --- |
| **Diagnosis** | Correct | False Positive | False Negative | Correct | False Positive | False Negative |  |
| Other benign mass | 8 | 7 | -- | 11 | 4 | -- |  |
| Endometrioma | 4 | 4 | -- | 8 | 0 | -- |  |
| Cystadenoma | 2 | 1 | -- | 2 | 1 | -- |  |
| Borderline tumors | 6 | 11 | -- | 14 | 3 | -- |  |
| **Total false positive rate** | **23/43 (53%)** | | | **8/43 (19%)** | | | **0**.**002** |
| Stage I/II Invasive Serous Adenocarcinoma | 18 | -- | 4 | 19 | -- | 3 |  |
| Stage III/IV Invasive Serous Adenocarcinoma | 21 | -- | 1 | 20 | -- | 2 |  |
| **Total false negative rate** | **5/44 (11%)** | | | **5/44 (11%)** | | | **1**.**0** |
| Stage I/II Invasive Clear Cell/Endometrioid Adenocarcinoma | 11 | -- | 7 | 14 | -- | 4 |  |
| Stage III/IV Invasive Clear Cell/Endometrioid Adenocarcinoma | 7 | -- | 1 | 8 | -- | 0 |  |
| **Total false negative rate** | **8/26 (31%)** | | | **4/26 (15%)** | | | **0**.**002** |
| Invasive cancer (other histologies) | 4 | -- | 3 | 5 | -- | 2 |  |
| **Total false negative rate** | **3/7 (43%)** | | | **2/7 (29%)** | | | **1**.**0** |
| Correct – accurate classification as benign, borderline or control versus invasive cancer  False positive – incorrectly assigned benign, borderline or control to invasive cancer  False negative – incorrectly assigned invasive cancer to benign, borderline or control | | | | | | |  |

**Supplementary File 2B Misclassification matrices for the neural network stratified by age.**

|  | **Cancer** | |  | **Benign/Borderline/Control** | |  | **Positive Predictive Value** | **Negative**  **Predictive**  **Value** |
| --- | --- | --- | --- | --- | --- | --- | --- | --- |
| **Age** | **Correct** | **False Negative** | **p-value^*^** | **Correct** | **False Positive** | **p-value^*^** |  |  |
| ≤ 51 years | 19 | 3 | 0.71 | 20 | 2 | 0.72 | 0.86 | 0.91 |
| > 51 years | 68 | 8 |  | 49 | 8 |  | 0.89 | 0.86 |
| **^*^**Fisher’s exact test (two-tailed) | | | | | | | | |

**Supplementary File 2C. miRNA expression by tumor histology and stage.**

|  | Advanced Endometrioid | | | Early Endometrioid | | |  |  |
| --- | --- | --- | --- | --- | --- | --- | --- | --- |
|  | Mean | St. Dev | N | Mean | St. dev | N | p (raw t.test) | Bonferroni-adjusted p |
| hsa-miR-23b-3p | 1.26 | 0.28 | 10 | 1.24 | 0.26 | 26 | 0.80 | 1.00 |
| hsa-miR-29a-3p | 2.68 | 0.31 | 10 | 2.44 | 0.39 | 26 | 0.08 | 1.00 |
| hsa-miR-32-5p | 1.71 | 0.57 | 10 | 1.36 | 0.40 | 26 | 0.05 | 0.66 |
| hsa-miR-92a-3p | 3.72 | 0.40 | 10 | 3.95 | 0.27 | 26 | 0.05 | 0.72 |
| hsa-miR-150-5p | 1.50 | 0.61 | 10 | 1.87 | 0.27 | 26 | 0.01 | 0.20 |
| hsa-miR-200a-3p | 1.51 | 0.83 | 10 | 1.64 | 0.53 | 26 | 0.58 | 1.00 |
| hsa-miR-200c-3p | 1.58 | 0.92 | 10 | 1.67 | 0.53 | 26 | 0.74 | 1.00 |
| hsa-miR-203a | 1.74 | 0.27 | 10 | 1.63 | 0.37 | 26 | 0.37 | 1.00 |
| hsa-miR-320c | 2.21 | 0.52 | 10 | 2.04 | 0.39 | 26 | 0.28 | 1.00 |
| hsa-miR-320d | 1.68 | 0.42 | 10 | 1.56 | 0.41 | 26 | 0.44 | 1.00 |
| hsa-miR-335-5p | 1.53 | 0.35 | 10 | 1.29 | 0.31 | 26 | 0.05 | 0.70 |
| hsa-miR-450b-5p | 1.57 | 0.40 | 10 | 1.36 | 0.34 | 26 | 0.11 | 1.00 |
| hsa-miR-1246 | 2.66 | 0.63 | 10 | 2.53 | 0.50 | 26 | 0.50 | 1.00 |
| hsa-miR-1307-5p | 1.59 | 0.19 | 10 | 1.59 | 0.27 | 26 | 0.99 | 1.00 |
|  |  |  |  |  |  |  |  |  |
|  | Advanced Serous | | | Early Serous | | |  |  |
|  | Mean | St. Dev | N | Mean | St. Dev | N | p (raw t.test) | Bonferroni-adjusted p |
| hsa-miR-23b-3p | 1.34 | 0.26 | 30 | 1.24 | 0.25 | 25 | 0.16 | 1.00 |
| hsa-miR-29a-3p | 2.50 | 0.32 | 30 | 2.55 | 0.37 | 25 | 0.64 | 1.00 |
| hsa-miR-32-5p | 1.45 | 0.52 | 30 | 1.29 | 0.45 | 25 | 0.24 | 1.00 |
| hsa-miR-92a-3p | 3.88 | 0.25 | 30 | 3.95 | 0.32 | 25 | 0.36 | 1.00 |
| hsa-miR-150-5p | 1.65 | 0.50 | 30 | 1.80 | 0.25 | 25 | 0.19 | 1.00 |
| hsa-miR-200a-3p | 1.22 | 0.59 | 30 | 1.38 | 0.50 | 25 | 0.30 | 1.00 |
| hsa-miR-200c-3p | 1.47 | 0.56 | 30 | 1.42 | 0.50 | 25 | 0.72 | 1.00 |
| hsa-miR-203a | 1.55 | 0.47 | 30 | 1.56 | 0.41 | 25 | 0.98 | 1.00 |
| hsa-miR-320c | 2.15 | 0.43 | 30 | 2.09 | 0.31 | 25 | 0.53 | 1.00 |
| hsa-miR-320d | 1.67 | 0.42 | 30 | 1.59 | 0.26 | 25 | 0.39 | 1.00 |
| hsa-miR-335-5p | 1.37 | 0.31 | 30 | 1.33 | 0.33 | 25 | 0.61 | 1.00 |
| hsa-miR-450b-5p | 1.35 | 0.37 | 30 | 1.19 | 0.38 | 25 | 0.13 | 1.00 |
| hsa-miR-1246 | 2.65 | 0.42 | 30 | 2.45 | 0.40 | 25 | 0.09 | 1.00 |
| hsa-miR-1307-5p | 1.60 | 0.20 | 30 | 1.57 | 0.16 | 25 | 0.62 | 1.00 |
|  |  |  |  |  |  |  |  |  |
|  | Advanced | | | Early | | |  |  |
|  | Mean | St. Dev | N | Mean | St. Dev | N | p (raw t.test) | Bonferroni-adjusted p |
| hsa-miR-23b-3p | 1.32 | 0.27 | 40 | 1.24 | 0.25 | 51 | 0.14 | 1.00 |
| hsa-miR-29a-3p | 2.55 | 0.33 | 40 | 2.49 | 0.38 | 51 | 0.45 | 1.00 |
| hsa-miR-32-5p | 1.51 | 0.54 | 40 | 1.33 | 0.42 | 51 | 0.07 | 0.96 |
| hsa-miR-92a-3p | 3.84 | 0.30 | 40 | 3.95 | 0.29 | 51 | 0.07 | 1.00 |
| hsa-miR-150-5p | 1.61 | 0.52 | 40 | 1.84 | 0.26 | 51 | 0.02 | 0.24 |
| hsa-miR-200a-3p | 1.29 | 0.66 | 40 | 1.51 | 0.53 | 51 | 0.08 | 1.00 |
| hsa-miR-200c-3p | 1.50 | 0.65 | 40 | 1.54 | 0.53 | 51 | 0.71 | 1.00 |
| hsa-miR-203a | 1.60 | 0.43 | 40 | 1.59 | 0.39 | 51 | 0.93 | 1.00 |
| hsa-miR-320c | 2.17 | 0.45 | 40 | 2.06 | 0.35 | 51 | 0.22 | 1.00 |
| hsa-miR-320d | 1.68 | 0.41 | 40 | 1.58 | 0.34 | 51 | 0.21 | 1.00 |
| hsa-miR-335-5p | 1.41 | 0.32 | 40 | 1.31 | 0.32 | 51 | 0.12 | 1.00 |
| hsa-miR-450b-5p | 1.40 | 0.39 | 40 | 1.27 | 0.36 | 51 | 0.11 | 1.00 |
| hsa-miR-1246 | 2.65 | 0.47 | 40 | 2.49 | 0.45 | 51 | 0.10 | 1.00 |
| hsa-miR-1307-5p | 1.60 | 0.20 | 40 | 1.58 | 0.22 | 51 | 0.73 | 1.00 |
