# Supplementary material for: Diagnostic potential for a serum miRNA neural network for detection of ovarian cancer
Source: eLife. 2017 Oct 31;6:e28932. doi: 10.7554/eLife.28932 (PMC5679755; doi:10.7554/eLife.28932)
Supplement: Supplementary file 3. — (A) Serum CA125 measurements among cancer and non-cancer cases in the two study populations. (B) Relationship between CA125 and miRNAs in the neural network. [file elife-28932-supp3.docx]

**Supplementary File 3A. Serum CA125 measurements among cancer and non-cancer cases in the two study populations.**

|  |  | N | Average | Standard deviation | Median | Q25 | Q75 | Z statistic | p-value† |
| --- | --- | --- | --- | --- | --- | --- | --- | --- | --- |
| NECC/PMP | Controls+Borderline | 22 | 121.19 | 194.02 | 42.00 | 10.50 | 118.00 | -2.97 | 0.003 |
|  | Cancer | 44 | 774.96 | 1591.19 | 128.50 | 31.30 | 624.50 |  |  |
| ERASMOS | Controls+Borderline | 21 | 132.40 | 193.27 | 45.00 | 16.10 | 172.70 | -3.29 | 0.001 |
|  | Cancer | 33 | 547.67 | 834.03 | 252.30 | 132.00 | 464.00 |  |  |
| Total |  | 120 | 480.15 | 1092.44 | 124.50 | 26.50 | 379.00 |  |  |
| †Mann-Whitney U-test  PMP – Pelvic Mass Protocol  NECC – New England Case Control Study  ERASMOS – Effects of Regional Analgesia on Serum miRNA after Oncology Surgery Study  BBC - Benign, Borderline, or Control  Q25 – 1^st^ quartile  Q75 – 2^nd^ quartile  The difference between cancer and non-cancer cases in the whole dataset was also statistically significant (Z=-4.33 p<0.0001) | | | | | | | | | |

**Supplementary File 3B. Relationship between CA-125 and miRNAs in the neural network**. Shown are the correlation coefficients between miRNAs selected using the fold change-based variable selection filter and CA-125 in patients with cancer and the Benign/Borderline/Control group.

|  | hsa-miR-23b-3p | hsa-miR-29a-3p | hsa-miR-32-5p | hsa-miR-92a-3p | hsa-miR-1246 |
| --- | --- | --- | --- | --- | --- |
| Cancer | -0.099 | -0.03 | 0.15 | -0.048 | -0.105 |
| N=77 | p=0.390 | p=0.794 | p=0.193 | p=0.681 | p=0.362 |
| BBC | -0.153 | 0.065 | 0.153 | 0.098 | -0.09 |
| N=43 | p=0.327 | p=0.681 | p=0.328 | p=0.532 | p=0.567 |
|  | hsa-miR-150-5p | hsa-miR-200a-3p | hsa-miR-200c-3p | hsa-miR-203a | hsa-miR-1307-5p |
| Cancer | -0.267 | 0.187 | 0.263 | 0.108 | -0.101 |
| N=77 | p=0.019 | p=0.103 | p=0.021 | p=0.348 | p=0.385 |
| BBC | -0.194 | 0.187 | 0.224 | 0.047 | 0.021 |
| N=43 | p=0.213 | p=0.229 | p=0.148 | p=0.764 | p=0.895 |
|  | hsa-miR-320c | hsa-miR-320d | hsa-miR-335-5p | hsa-miR-450b-5p |  |
| Cancer | -0.103 | -0.098 | 0.099 | -0.007 |  |
| N=77 | p=0.374 | p=0.396 | p=0.393 | p=0.951 |  |
| BBC | 0.107 | -0.023 | 0.132 | -0.12 |  |
| N=43 | p=0.495 | p=0.883 | p=0.399 | p=0.444 |  |
| BBC - Benign, Borderline, or Control | | | | | |
