# Supplementary material for: Diagnostic potential for a serum miRNA neural network for detection of ovarian cancer
Source: eLife. 2017 Oct 31;6:e28932. doi: 10.7554/eLife.28932 (PMC5679755; doi:10.7554/eLife.28932)
Supplement: Supplementary file 4. — (A) Mapping of the 14-miRNA dataset from the miRNA-sequencing study onto the GSE31568 dataset published by Keller, et al. (B) Comparison of the neural network (NN) classifier with the tissue-based MiROvaR signature by Bagnoli et al. [file elife-28932-supp4.docx]

**Supplementary file 4A. Mapping of the 14-miRNA dataset from the miRNA-sequencing study onto the GSE31568 dataset published by Keller, *et al* (11).** All of the targets of interest were contained in both datasets.

| Dataset | Present study | GSE31568, Keller *et al(11).* |  |  |
| --- | --- | --- | --- | --- |
| Platform | Exiqon Human panels I and II | febit Homo Sapiens miRBase 13.0 | Present in Keller *et al.* set | Notes |
|  | hsa-miR-1246 | hsa-miR-1246 | + |  |
|  | hsa-miR-1307-5p | hsa-miR-1307 | + |  |
|  | hsa-miR-150-5p | hsa-miR-150 | + |  |
|  | hsa-miR-200a-3p | hsa-miR-200a* | + |  |
|  | hsa-miR-200c-3p | hsa-miR-200c* | + |  |
|  | hsa-miR-203a | hsa-miR-203 | + | Strand unspecified |
|  | hsa-miR-23b-3p | hsa-miR-23b* | + |  |
|  | hsa-miR-29a-3p | hsa-miR-29a* | + |  |
|  | hsa-miR-320c | hsa-miR-32 | + |  |
|  | hsa-miR-320d | hsa-miR-320c | + |  |
|  | hsa-miR-32-5p | hsa-miR-320d | + |  |
|  | hsa-miR-335-5p | hsa-miR-335 | + |  |
|  | hsa-miR-450b-5p | hsa-miR-450b-3p | + |  |
|  | hsa-miR-92a-3p | hsa-miR-92a | + | Strand unspecified |

**Supplementary file 4B. Comparision of the neural network (NN) classifier with the tissue-based MiROvaR signature by Bagnoli *et al* (9)*.*** Notably the tissue signature was modeled on predicting prognosis, not diagnosis, which accounts for some of the difference. In addition, about two-thirds of the miRNAs in the tissue signature are not reliably detectable in circulation, which can be attributed to the fact that only a small fraction of miRNAs circulate in serum.

| MirOvaR | Detected in the filtered set (>10 in at least 50% of samples in both ERASMOS and PMP/NECC) | Present in NN classifier | Detected in at least 1 case in the present work |
| --- | --- | --- | --- |
| miR-890 |  |  |  |
| miR-513a-5p |  |  |  |
| miR-513b-5p |  |  |  |
| miR-135b-5p |  |  | *+* |
| miR-141-3p |  |  | *+* |
| miR-200c-3p | *+* | *+* | *+* |
| miR-429 |  |  | *+* |
| miR-200a-3p | *+* | *+* | *+* |
| miR-200b-3p |  |  | *+* |
| miR-592 |  |  |  |
| miR-508-3p |  |  | *+* |
| miR-514a-3p |  |  | *+* |
| miR-509-3p |  |  | *+* |
| miR-509-5p |  |  |  |
| miR-506-3p |  |  |  |
| miR-507 |  |  |  |
| miR-143-5p |  |  | *+* |
| miR-486-5p | *+* |  | *+* |
| miR-195-3p |  |  | *+* |
| miR-23a-5p |  |  | *+* |
| miR-193b-5p |  |  | *+* |
| miR-574-5p | *+* |  | *+* |
| miR-99b-5p | *+* |  | *+* |
| miR-151a-3p | *+* |  | *+* |
| miR-30d-5p | *+* |  | *+* |
| miR-423-5p | *+* |  | *+* |
| miR-30b-3p |  |  | *+* |
| miR-330-3p | *+* |  | *+* |
| miR-452-5p |  |  | *+* |
| miR-100-3p |  |  |  |
| miR-193a-5p | *+* |  | *+* |
| miR-29c-5p |  |  | *+* |
| miR-29a-5p |  |  |  |
| miR-484 | *+* |  | *+* |
| miR-769-5p | *+* |  | *+* |
