# Supplementary material for: Diagnostic potential for a serum miRNA neural network for detection of ovarian cancer
Source: eLife. 2017 Oct 31;6:e28932. doi: 10.7554/eLife.28932 (PMC5679755; doi:10.7554/eLife.28932)
Supplement: Supplementary file 5. [file elife-28932-supp5.docx]

**Supplementary File 5. Univariate comparison of miRNA average expression values between patients with cancer and patients in the benign/borderline/control group.**

| miRNA | Cancer Average (n=98) | Cancer  SD (n=98) | BBC  Average  (n=81) | BBC  SD (n=81) | df | t | Raw  p-value | FDR |
| --- | --- | --- | --- | --- | --- | --- | --- | --- |
| hsa-miR-200c-3p | 1.51 | 0.57 | 1.08 | 0.38 | 177 | 5.712 | 4.64E-08 | 8.91E-06 |
| hsa-miR-320d | 1.60 | 0.38 | 1.37 | 0.23 | 177 | 4.917 | 2.00E-06 | 1.92E-04 |
| hsa-miR-320c | 2.09 | 0.40 | 1.89 | 0.24 | 177 | 4.021 | 8.60E-05 | 4.51E-03 |
| hsa-miR-1246 | 2.56 | 0.49 | 2.25 | 0.51 | 177 | 3.997 | 9.40E-05 | 4.51E-03 |
| hsa-miR-29a-3p | 2.51 | 0.35 | 2.33 | 0.28 | 177 | 3.592 | 4.25E-04 | 1.60E-02 |
| hsa-miR-200a-3p | 1.39 | 0.60 | 1.11 | 0.42 | 177 | 3.546 | 5.01E-04 | 1.60E-02 |
| hsa-miR-203a | 1.59 | 0.41 | 1.39 | 0.40 | 177 | 3.273 | 1.28E-03 | 3.51E-02 |
| hsa-miR-450b-5p | 1.33 | 0.38 | 1.16 | 0.38 | 177 | 3.118 | 2.12E-03 | 4.46E-02 |
| hsa-miR-1307-5p | 1.58 | 0.21 | 1.49 | 0.19 | 177 | 3.106 | 2.21E-03 | 4.46E-02 |
| hsa-miR-30d-5p | 3.77 | 0.22 | 3.86 | 0.17 | 177 | -3.064 | 2.53E-03 | 4.46E-02 |
| hsa-miR-486-3p | 2.35 | 0.27 | 2.47 | 0.25 | 177 | -3.060 | 2.56E-03 | 4.46E-02 |
| hsa-miR-425-5p | 2.28 | 0.21 | 2.37 | 0.20 | 177 | -2.824 | 5.29E-03 | 8.17E-02 |
| hsa-miR-320b | 2.68 | 0.31 | 2.57 | 0.21 | 177 | 2.809 | 5.53E-03 | 8.17E-02 |
| hsa-miR-92a-3p | 3.90 | 0.30 | 4.02 | 0.29 | 177 | -2.749 | 6.59E-03 | 9.04E-02 |
| hsa-miR-181b-5p | 2.09 | 0.22 | 2.00 | 0.19 | 177 | 2.720 | 7.18E-03 | 9.18E-02 |
| hsa-miR-181d-5p | 1.28 | 0.26 | 1.18 | 0.27 | 177 | 2.528 | 1.23E-02 | 1.48E-01 |
| hsa-miR-21-5p | 3.92 | 0.27 | 3.82 | 0.26 | 177 | 2.473 | 1.43E-02 | 1.62E-01 |
| hsa-miR-339-3p | 1.46 | 0.21 | 1.52 | 0.17 | 177 | -2.386 | 1.81E-02 | 1.74E-01 |
| hsa-miR-145-3p | 1.81 | 0.26 | 1.72 | 0.27 | 177 | 2.381 | 1.83E-02 | 1.74E-01 |
| hsa-miR-23b-3p | 1.28 | 0.26 | 1.19 | 0.22 | 177 | 2.379 | 1.84E-02 | 1.74E-01 |
| hsa-miR-7706 | 1.18 | 0.32 | 1.29 | 0.29 | 177 | -2.366 | 1.91E-02 | 1.74E-01 |
| hsa-miR-128-3p | 2.92 | 0.30 | 3.01 | 0.22 | 177 | -2.250 | 2.57E-02 | 2.16E-01 |
| hsa-miR-361-5p | 1.47 | 0.22 | 1.40 | 0.20 | 177 | 2.247 | 2.59E-02 | 2.16E-01 |
| hsa-miR-335-5p | 1.35 | 0.32 | 1.25 | 0.30 | 177 | 2.177 | 3.08E-02 | 2.32E-01 |
| hsa-miR-484 | 2.48 | 0.21 | 2.54 | 0.19 | 177 | -2.154 | 3.26E-02 | 2.32E-01 |
| hsa-miR-142-3p | 1.86 | 0.35 | 1.75 | 0.38 | 177 | 2.154 | 3.26E-02 | 2.32E-01 |
| hsa-let-7b-3p | 1.18 | 0.26 | 1.26 | 0.23 | 177 | -2.153 | 3.27E-02 | 2.32E-01 |
| hsa-miR-16-2-3p | 3.05 | 0.33 | 3.16 | 0.32 | 177 | -2.114 | 3.59E-02 | 2.40E-01 |
| hsa-miR-3158-3p | 1.55 | 0.34 | 1.66 | 0.35 | 177 | -2.102 | 3.70E-02 | 2.40E-01 |
| hsa-miR-532-5p | 2.79 | 0.24 | 2.86 | 0.27 | 177 | -2.096 | 3.75E-02 | 2.40E-01 |
| hsa-miR-150-5p | 1.74 | 0.41 | 1.86 | 0.34 | 177 | -2.080 | 3.90E-02 | 2.41E-01 |
| hsa-miR-409-3p | 2.16 | 0.51 | 2.31 | 0.47 | 177 | -2.061 | 4.08E-02 | 2.45E-01 |
| hsa-miR-483-5p | 1.20 | 0.67 | 1.00 | 0.59 | 177 | 2.025 | 4.44E-02 | 2.52E-01 |
| hsa-miR-27a-3p | 3.13 | 0.28 | 3.05 | 0.28 | 177 | 2.023 | 4.46E-02 | 2.52E-01 |
| hsa-miR-654-3p | 1.46 | 0.44 | 1.59 | 0.44 | 177 | -1.992 | 4.79E-02 | 2.63E-01 |
| hsa-miR-486-5p | 4.53 | 0.30 | 4.62 | 0.32 | 177 | -1.963 | 5.12E-02 | 2.73E-01 |
| hsa-miR-181a-2-3p | 1.61 | 0.21 | 1.67 | 0.19 | 177 | -1.916 | 5.70E-02 | 2.93E-01 |
| hsa-miR-543 | 1.59 | 0.51 | 1.74 | 0.49 | 177 | -1.896 | 5.95E-02 | 2.93E-01 |
| hsa-miR-4443 | 1.23 | 0.33 | 1.13 | 0.35 | 177 | 1.895 | 5.98E-02 | 2.93E-01 |
| hsa-miR-4732-5p | 1.52 | 0.38 | 1.62 | 0.37 | 177 | -1.881 | 6.17E-02 | 2.93E-01 |
| hsa-miR-323b-3p | 1.21 | 0.49 | 1.35 | 0.52 | 177 | -1.873 | 6.27E-02 | 2.93E-01 |
| hsa-miR-584-5p | 2.57 | 0.19 | 2.62 | 0.18 | 177 | -1.847 | 6.64E-02 | 3.04E-01 |
| hsa-miR-2110 | 1.93 | 0.20 | 1.99 | 0.17 | 177 | -1.810 | 7.20E-02 | 3.10E-01 |
| hsa-miR-25-3p | 3.74 | 0.30 | 3.82 | 0.32 | 177 | -1.810 | 7.21E-02 | 3.10E-01 |
| hsa-miR-30e-3p | 2.30 | 0.19 | 2.25 | 0.23 | 177 | 1.806 | 7.26E-02 | 3.10E-01 |
| hsa-miR-1180-3p | 1.82 | 0.31 | 1.91 | 0.32 | 177 | -1.782 | 7.64E-02 | 3.14E-01 |
| hsa-miR-140-3p | 3.00 | 0.28 | 3.08 | 0.30 | 177 | -1.780 | 7.68E-02 | 3.14E-01 |
| hsa-miR-27b-3p | 2.78 | 0.24 | 2.72 | 0.23 | 177 | 1.731 | 8.51E-02 | 3.37E-01 |
| hsa-miR-24-3p | 3.19 | 0.22 | 3.13 | 0.23 | 177 | 1.726 | 8.61E-02 | 3.37E-01 |
| hsa-miR-338-5p | 1.76 | 0.30 | 1.67 | 0.36 | 177 | 1.694 | 9.21E-02 | 3.54E-01 |
| hsa-miR-32-5p | 1.41 | 0.49 | 1.29 | 0.39 | 177 | 1.680 | 9.47E-02 | 3.56E-01 |
| hsa-miR-125a-5p | 2.02 | 0.48 | 2.14 | 0.45 | 177 | -1.671 | 9.65E-02 | 3.56E-01 |
| hsa-miR-451a | 4.58 | 0.35 | 4.67 | 0.38 | 177 | -1.652 | 1.00E-01 | 3.64E-01 |
| hsa-miR-185-3p | 1.12 | 0.26 | 1.18 | 0.20 | 177 | -1.641 | 1.03E-01 | 3.65E-01 |
| hsa-miR-130a-3p | 1.21 | 0.38 | 1.13 | 0.31 | 177 | 1.618 | 1.07E-01 | 3.75E-01 |
| hsa-let-7b-5p | 3.97 | 0.27 | 4.03 | 0.29 | 177 | -1.586 | 1.15E-01 | 3.93E-01 |
| hsa-miR-501-3p | 2.11 | 0.27 | 2.17 | 0.29 | 177 | -1.577 | 1.17E-01 | 3.93E-01 |
| hsa-miR-382-5p | 1.63 | 0.37 | 1.72 | 0.43 | 177 | -1.548 | 1.23E-01 | 4.02E-01 |
| hsa-miR-98-5p | 2.09 | 0.27 | 2.03 | 0.27 | 177 | 1.542 | 1.25E-01 | 4.02E-01 |
| hsa-let-7d-3p | 2.63 | 0.26 | 2.69 | 0.25 | 177 | -1.532 | 1.27E-01 | 4.02E-01 |
| hsa-miR-148a-3p | 4.17 | 0.27 | 4.10 | 0.28 | 177 | 1.531 | 1.28E-01 | 4.02E-01 |
| hsa-miR-328-3p | 1.76 | 0.37 | 1.84 | 0.38 | 177 | -1.509 | 1.33E-01 | 4.12E-01 |
| hsa-miR-16-5p | 2.54 | 0.37 | 2.63 | 0.36 | 177 | -1.488 | 1.39E-01 | 4.22E-01 |
| hsa-miR-432-5p | 1.74 | 0.45 | 1.85 | 0.47 | 177 | -1.462 | 1.45E-01 | 4.30E-01 |
| hsa-miR-942-5p | 1.27 | 0.24 | 1.32 | 0.25 | 177 | -1.462 | 1.45E-01 | 4.30E-01 |
| hsa-miR-340-5p | 2.28 | 0.33 | 2.21 | 0.32 | 177 | 1.441 | 1.51E-01 | 4.40E-01 |
| hsa-miR-106b-3p | 2.49 | 0.23 | 2.54 | 0.24 | 177 | -1.425 | 1.56E-01 | 4.47E-01 |
| hsa-miR-24-2-5p | 1.34 | 0.24 | 1.29 | 0.25 | 177 | 1.390 | 1.66E-01 | 4.69E-01 |
| hsa-miR-30a-3p | 1.65 | 0.24 | 1.60 | 0.24 | 177 | 1.352 | 1.78E-01 | 4.95E-01 |
| hsa-miR-143-3p | 3.11 | 0.31 | 3.05 | 0.34 | 177 | 1.330 | 1.85E-01 | 5.04E-01 |
| hsa-miR-423-5p | 4.02 | 0.32 | 4.09 | 0.32 | 177 | -1.327 | 1.86E-01 | 5.04E-01 |
| hsa-miR-7-5p | 2.55 | 0.28 | 2.50 | 0.25 | 177 | 1.300 | 1.95E-01 | 5.17E-01 |
| hsa-miR-26a-5p | 3.77 | 0.24 | 3.72 | 0.28 | 177 | 1.296 | 1.97E-01 | 5.17E-01 |
| hsa-miR-361-3p | 2.37 | 0.17 | 2.40 | 0.17 | 177 | -1.274 | 2.04E-01 | 5.30E-01 |
| hsa-miR-152-3p | 1.83 | 0.22 | 1.78 | 0.25 | 177 | 1.260 | 2.09E-01 | 5.36E-01 |
| hsa-miR-3615 | 2.56 | 0.21 | 2.60 | 0.19 | 177 | -1.248 | 2.14E-01 | 5.40E-01 |
| hsa-miR-191-5p | 3.13 | 0.19 | 3.17 | 0.18 | 177 | -1.239 | 2.17E-01 | 5.41E-01 |
| hsa-let-7e-5p | 1.93 | 0.26 | 1.88 | 0.32 | 177 | 1.211 | 2.27E-01 | 5.54E-01 |
| hsa-miR-130b-5p | 1.14 | 0.51 | 1.23 | 0.50 | 177 | -1.200 | 2.32E-01 | 5.54E-01 |
| hsa-miR-93-5p | 2.34 | 0.27 | 2.38 | 0.23 | 177 | -1.200 | 2.32E-01 | 5.54E-01 |
| hsa-miR-374a-5p | 1.21 | 0.40 | 1.14 | 0.33 | 177 | 1.195 | 2.34E-01 | 5.54E-01 |
| hsa-miR-151a-3p | 3.34 | 0.22 | 3.38 | 0.22 | 177 | -1.167 | 2.45E-01 | 5.73E-01 |
| hsa-miR-345-5p | 1.35 | 0.24 | 1.39 | 0.19 | 177 | -1.157 | 2.49E-01 | 5.76E-01 |
| hsa-miR-450a-5p | 1.36 | 0.35 | 1.30 | 0.39 | 177 | 1.143 | 2.55E-01 | 5.82E-01 |
| hsa-miR-139-3p | 1.22 | 0.40 | 1.28 | 0.38 | 177 | -1.128 | 2.61E-01 | 5.89E-01 |
| hsa-miR-30e-5p | 2.84 | 0.27 | 2.89 | 0.28 | 177 | -1.116 | 2.66E-01 | 5.94E-01 |
| hsa-miR-15b-5p | 1.53 | 0.38 | 1.60 | 0.37 | 177 | -1.096 | 2.75E-01 | 6.06E-01 |
| hsa-miR-550a-5p | 1.17 | 0.26 | 1.21 | 0.24 | 177 | -1.082 | 2.81E-01 | 6.08E-01 |
| hsa-miR-197-3p | 1.25 | 0.34 | 1.30 | 0.32 | 177 | -1.079 | 2.82E-01 | 6.08E-01 |
| hsa-miR-375 | 1.89 | 0.52 | 1.98 | 0.48 | 177 | -1.058 | 2.91E-01 | 6.18E-01 |
| hsa-miR-199b-3p | 1.93 | 0.28 | 1.89 | 0.29 | 177 | 1.038 | 3.01E-01 | 6.18E-01 |
| hsa-miR-342-5p | 1.79 | 0.21 | 1.82 | 0.23 | 177 | -1.033 | 3.03E-01 | 6.18E-01 |
| hsa-miR-20b-5p | 1.34 | 0.40 | 1.40 | 0.33 | 177 | -1.031 | 3.04E-01 | 6.18E-01 |
| hsa-miR-363-3p | 2.62 | 0.29 | 2.67 | 0.28 | 177 | -1.022 | 3.08E-01 | 6.18E-01 |
| hsa-miR-20a-5p | 2.38 | 0.35 | 2.42 | 0.29 | 177 | -1.012 | 3.13E-01 | 6.18E-01 |
| hsa-miR-125b-5p | 1.75 | 0.41 | 1.82 | 0.42 | 177 | -1.012 | 3.13E-01 | 6.18E-01 |
| hsa-miR-15b-3p | 1.41 | 0.34 | 1.46 | 0.41 | 177 | -1.007 | 3.15E-01 | 6.18E-01 |
| hsa-miR-335-3p | 1.31 | 0.41 | 1.37 | 0.38 | 177 | -1.007 | 3.15E-01 | 6.18E-01 |
| hsa-miR-99b-5p | 2.47 | 0.30 | 2.42 | 0.33 | 177 | 0.981 | 3.28E-01 | 6.21E-01 |
| hsa-miR-576-3p | 1.37 | 0.35 | 1.32 | 0.34 | 177 | 0.973 | 3.32E-01 | 6.21E-01 |
| hsa-miR-941 | 2.05 | 0.22 | 2.09 | 0.23 | 177 | -0.962 | 3.38E-01 | 6.21E-01 |
| hsa-miR-148a-5p | 1.36 | 0.25 | 1.32 | 0.32 | 177 | 0.957 | 3.40E-01 | 6.21E-01 |
| hsa-miR-144-3p | 2.25 | 0.52 | 2.18 | 0.46 | 177 | 0.956 | 3.40E-01 | 6.21E-01 |
| hsa-miR-186-5p | 2.59 | 0.28 | 2.64 | 0.30 | 177 | -0.953 | 3.42E-01 | 6.21E-01 |
| hsa-miR-10a-5p | 3.21 | 0.38 | 3.16 | 0.29 | 177 | 0.947 | 3.45E-01 | 6.21E-01 |
| hsa-miR-199a-3p | 2.02 | 0.28 | 1.98 | 0.28 | 177 | 0.943 | 3.47E-01 | 6.21E-01 |
| hsa-miR-26b-5p | 2.74 | 0.33 | 2.70 | 0.28 | 177 | 0.925 | 3.56E-01 | 6.21E-01 |
| hsa-miR-126-3p | 3.52 | 0.23 | 3.48 | 0.28 | 177 | 0.924 | 3.57E-01 | 6.21E-01 |
| hsa-miR-19b-3p | 1.72 | 0.35 | 1.77 | 0.39 | 177 | -0.923 | 3.57E-01 | 6.21E-01 |
| hsa-miR-134-5p | 2.02 | 0.35 | 2.07 | 0.45 | 177 | -0.922 | 3.58E-01 | 6.21E-01 |
| hsa-let-7f-5p | 3.74 | 0.29 | 3.70 | 0.28 | 177 | 0.909 | 3.64E-01 | 6.21E-01 |
| hsa-miR-23a-3p | 2.11 | 0.25 | 2.07 | 0.25 | 177 | 0.908 | 3.65E-01 | 6.21E-01 |
| hsa-miR-194-5p | 1.93 | 0.30 | 1.97 | 0.30 | 177 | -0.902 | 3.68E-01 | 6.21E-01 |
| hsa-miR-30a-5p | 2.42 | 0.28 | 2.39 | 0.26 | 177 | 0.901 | 3.69E-01 | 6.21E-01 |
| hsa-miR-1307-3p | 2.37 | 0.24 | 2.40 | 0.21 | 177 | -0.882 | 3.79E-01 | 6.33E-01 |
| hsa-miR-142-5p | 3.07 | 0.30 | 3.11 | 0.29 | 177 | -0.866 | 3.88E-01 | 6.41E-01 |
| hsa-miR-28-5p | 1.21 | 0.25 | 1.17 | 0.30 | 177 | 0.860 | 3.91E-01 | 6.41E-01 |
| hsa-miR-223-5p | 2.83 | 0.27 | 2.79 | 0.30 | 177 | 0.846 | 3.98E-01 | 6.48E-01 |
| hsa-miR-424-3p | 1.61 | 0.23 | 1.64 | 0.22 | 177 | -0.815 | 4.16E-01 | 6.71E-01 |
| hsa-miR-320a | 3.85 | 0.23 | 3.82 | 0.20 | 177 | 0.786 | 4.33E-01 | 6.91E-01 |
| hsa-miR-148b-3p | 2.78 | 0.22 | 2.76 | 0.20 | 177 | 0.781 | 4.36E-01 | 6.91E-01 |
| hsa-miR-625-3p | 1.86 | 0.25 | 1.83 | 0.22 | 177 | 0.765 | 4.45E-01 | 6.91E-01 |
| hsa-miR-19a-3p | 1.47 | 0.36 | 1.51 | 0.34 | 177 | -0.763 | 4.46E-01 | 6.91E-01 |
| hsa-miR-744-5p | 2.37 | 0.22 | 2.40 | 0.24 | 177 | -0.761 | 4.48E-01 | 6.91E-01 |
| hsa-miR-370-3p | 1.56 | 0.42 | 1.61 | 0.49 | 177 | -0.757 | 4.50E-01 | 6.91E-01 |
| hsa-miR-139-5p | 1.78 | 0.31 | 1.82 | 0.30 | 177 | -0.724 | 4.70E-01 | 7.16E-01 |
| hsa-miR-222-3p | 2.22 | 0.17 | 2.24 | 0.15 | 177 | -0.718 | 4.74E-01 | 7.16E-01 |
| hsa-miR-221-3p | 2.48 | 0.20 | 2.46 | 0.20 | 177 | 0.652 | 5.15E-01 | 7.73E-01 |
| hsa-miR-660-5p | 1.46 | 0.33 | 1.49 | 0.31 | 177 | -0.633 | 5.27E-01 | 7.79E-01 |
| hsa-miR-193a-5p | 1.84 | 0.28 | 1.81 | 0.27 | 177 | 0.627 | 5.31E-01 | 7.79E-01 |
| hsa-miR-1285-3p | 1.05 | 0.32 | 1.08 | 0.30 | 177 | -0.624 | 5.34E-01 | 7.79E-01 |
| hsa-miR-629-5p | 2.73 | 0.25 | 2.70 | 0.24 | 177 | 0.621 | 5.35E-01 | 7.79E-01 |
| hsa-miR-103a-3p | 2.64 | 0.31 | 2.62 | 0.28 | 177 | 0.611 | 5.42E-01 | 7.82E-01 |
| hsa-miR-423-3p | 3.15 | 0.14 | 3.16 | 0.16 | 177 | -0.583 | 5.61E-01 | 8.03E-01 |
| hsa-miR-340-3p | 1.14 | 0.29 | 1.11 | 0.33 | 177 | 0.578 | 5.64E-01 | 8.03E-01 |
| hsa-miR-323a-3p | 1.07 | 0.47 | 1.11 | 0.50 | 177 | -0.565 | 5.73E-01 | 8.08E-01 |
| hsa-miR-378c | 1.34 | 0.27 | 1.31 | 0.27 | 177 | 0.558 | 5.78E-01 | 8.10E-01 |
| hsa-miR-122-5p | 3.86 | 0.51 | 3.90 | 0.45 | 177 | -0.542 | 5.88E-01 | 8.19E-01 |
| hsa-miR-155-5p | 1.78 | 0.22 | 1.76 | 0.22 | 177 | 0.533 | 5.94E-01 | 8.21E-01 |
| hsa-miR-664a-5p | 1.48 | 0.18 | 1.46 | 0.18 | 177 | 0.520 | 6.04E-01 | 8.28E-01 |
| hsa-miR-574-5p | 1.67 | 0.31 | 1.69 | 0.30 | 177 | -0.503 | 6.16E-01 | 8.30E-01 |
| hsa-miR-99a-5p | 3.22 | 0.30 | 3.24 | 0.27 | 177 | -0.502 | 6.17E-01 | 8.30E-01 |
| hsa-miR-1908-5p | 1.03 | 0.26 | 1.05 | 0.27 | 177 | -0.498 | 6.19E-01 | 8.30E-01 |
| hsa-miR-6842-3p | 1.25 | 0.18 | 1.23 | 0.23 | 177 | 0.477 | 6.34E-01 | 8.30E-01 |
| hsa-miR-192-5p | 2.70 | 0.24 | 2.72 | 0.27 | 177 | -0.475 | 6.36E-01 | 8.30E-01 |
| hsa-miR-22-5p | 1.50 | 0.25 | 1.48 | 0.25 | 177 | 0.474 | 6.36E-01 | 8.30E-01 |
| hsa-let-7g-5p | 3.49 | 0.28 | 3.47 | 0.25 | 177 | 0.464 | 6.43E-01 | 8.30E-01 |
| hsa-miR-10b-5p | 3.45 | 0.37 | 3.42 | 0.31 | 177 | 0.457 | 6.48E-01 | 8.30E-01 |
| hsa-miR-107 | 1.89 | 0.34 | 1.91 | 0.32 | 177 | -0.450 | 6.53E-01 | 8.30E-01 |
| hsa-miR-146a-5p | 3.24 | 0.24 | 3.26 | 0.25 | 177 | -0.447 | 6.55E-01 | 8.30E-01 |
| hsa-miR-17-5p | 2.00 | 0.29 | 2.02 | 0.23 | 177 | -0.441 | 6.60E-01 | 8.30E-01 |
| hsa-miR-503-5p | 1.19 | 0.29 | 1.21 | 0.26 | 177 | -0.428 | 6.69E-01 | 8.30E-01 |
| hsa-miR-146b-5p | 2.92 | 0.21 | 2.90 | 0.21 | 177 | 0.426 | 6.71E-01 | 8.30E-01 |
| hsa-miR-652-3p | 1.74 | 0.29 | 1.76 | 0.26 | 177 | -0.419 | 6.76E-01 | 8.30E-01 |
| hsa-miR-181a-5p | 2.89 | 0.16 | 2.90 | 0.16 | 177 | -0.413 | 6.80E-01 | 8.30E-01 |
| hsa-miR-185-5p | 2.96 | 0.37 | 2.98 | 0.33 | 177 | -0.410 | 6.82E-01 | 8.30E-01 |
| hsa-miR-589-5p | 1.34 | 0.20 | 1.33 | 0.19 | 177 | 0.397 | 6.91E-01 | 8.30E-01 |
| hsa-miR-769-5p | 1.27 | 0.26 | 1.29 | 0.28 | 177 | -0.397 | 6.92E-01 | 8.30E-01 |
| hsa-miR-182-5p | 2.52 | 0.31 | 2.50 | 0.31 | 177 | 0.396 | 6.92E-01 | 8.30E-01 |
| hsa-miR-144-5p | 1.84 | 0.44 | 1.86 | 0.36 | 177 | -0.396 | 6.93E-01 | 8.30E-01 |
| hsa-miR-1301-3p | 1.54 | 0.27 | 1.55 | 0.25 | 177 | -0.392 | 6.96E-01 | 8.30E-01 |
| hsa-miR-100-5p | 2.61 | 0.30 | 2.60 | 0.32 | 177 | 0.363 | 7.17E-01 | 8.46E-01 |
| hsa-miR-215-5p | 1.53 | 0.35 | 1.51 | 0.39 | 177 | 0.362 | 7.18E-01 | 8.46E-01 |
| hsa-miR-3613-5p | 1.26 | 0.35 | 1.24 | 0.31 | 177 | 0.356 | 7.22E-01 | 8.46E-01 |
| hsa-miR-22-3p | 3.39 | 0.22 | 3.40 | 0.20 | 177 | -0.347 | 7.29E-01 | 8.49E-01 |
| hsa-miR-330-3p | 1.57 | 0.24 | 1.56 | 0.28 | 177 | 0.336 | 7.37E-01 | 8.53E-01 |
| hsa-miR-381-3p | 1.48 | 0.40 | 1.51 | 0.51 | 177 | -0.330 | 7.42E-01 | 8.53E-01 |
| hsa-let-7a-3p | 1.71 | 0.27 | 1.70 | 0.27 | 177 | 0.309 | 7.58E-01 | 8.66E-01 |
| hsa-miR-28-3p | 2.33 | 0.18 | 2.32 | 0.20 | 177 | 0.302 | 7.63E-01 | 8.66E-01 |
| hsa-miR-4433b-3p | 1.72 | 0.48 | 1.74 | 0.54 | 177 | -0.291 | 7.71E-01 | 8.71E-01 |
| hsa-let-7i-5p | 3.90 | 0.24 | 3.89 | 0.22 | 177 | 0.277 | 7.82E-01 | 8.73E-01 |
| hsa-miR-140-5p | 1.71 | 0.29 | 1.73 | 0.27 | 177 | -0.274 | 7.84E-01 | 8.73E-01 |
| hsa-miR-502-3p | 1.36 | 0.27 | 1.37 | 0.26 | 177 | -0.271 | 7.87E-01 | 8.73E-01 |
| hsa-miR-425-3p | 1.52 | 0.22 | 1.53 | 0.19 | 177 | -0.257 | 7.98E-01 | 8.80E-01 |
| hsa-miR-493-5p | 1.15 | 0.43 | 1.17 | 0.51 | 177 | -0.240 | 8.10E-01 | 8.89E-01 |
| hsa-miR-199a-5p | 2.15 | 0.31 | 2.14 | 0.32 | 177 | 0.223 | 8.24E-01 | 8.92E-01 |
| hsa-let-7a-5p | 3.84 | 0.28 | 3.83 | 0.25 | 177 | 0.222 | 8.25E-01 | 8.92E-01 |
| hsa-miR-500a-3p | 1.13 | 0.28 | 1.14 | 0.29 | 177 | -0.219 | 8.27E-01 | 8.92E-01 |
| hsa-miR-378a-3p | 2.76 | 0.19 | 2.75 | 0.21 | 177 | 0.208 | 8.36E-01 | 8.97E-01 |
| hsa-miR-30c-5p | 2.42 | 0.22 | 2.42 | 0.23 | 177 | 0.150 | 8.81E-01 | 9.39E-01 |
| hsa-miR-106b-5p | 1.63 | 0.37 | 1.63 | 0.34 | 177 | 0.139 | 8.89E-01 | 9.43E-01 |
| hsa-miR-183-5p | 2.42 | 0.29 | 2.41 | 0.31 | 177 | 0.101 | 9.19E-01 | 9.70E-01 |
| hsa-miR-223-3p | 2.08 | 0.37 | 2.08 | 0.39 | 177 | -0.094 | 9.25E-01 | 9.71E-01 |
| hsa-miR-126-5p | 2.03 | 0.27 | 2.03 | 0.29 | 177 | 0.084 | 9.33E-01 | 9.72E-01 |
| hsa-miR-92b-3p | 1.53 | 0.39 | 1.54 | 0.31 | 177 | -0.079 | 9.37E-01 | 9.72E-01 |
| hsa-miR-224-5p | 1.31 | 0.41 | 1.32 | 0.35 | 177 | -0.062 | 9.51E-01 | 9.81E-01 |
| hsa-let-7d-5p | 2.82 | 0.27 | 2.82 | 0.24 | 177 | 0.051 | 9.59E-01 | 9.84E-01 |
| hsa-let-7c-5p | 2.57 | 0.22 | 2.58 | 0.20 | 177 | -0.046 | 9.63E-01 | 9.84E-01 |
| hsa-miR-1 | 1.42 | 0.44 | 1.42 | 0.55 | 177 | -0.039 | 9.69E-01 | 9.85E-01 |
| hsa-miR-421 | 1.10 | 0.27 | 1.10 | 0.25 | 177 | -0.031 | 9.75E-01 | 9.85E-01 |
| hsa-miR-101-3p | 3.25 | 0.38 | 3.25 | 0.35 | 177 | -0.022 | 9.83E-01 | 9.88E-01 |
| hsa-miR-379-5p | 1.37 | 0.39 | 1.37 | 0.52 | 177 | 0.004 | 9.97E-01 | 9.97E-01 |
