# Supplementary material for: Diagnostic potential for a serum miRNA neural network for detection of ovarian cancer
Source: eLife. 2017 Oct 31;6:e28932. doi: 10.7554/eLife.28932 (PMC5679755; doi:10.7554/eLife.28932)
Supplement: Transparent reporting form [file elife-28932-transrepform.docx]

***eLife’s* transparent reporting form**

We encourage authors to provide detailed information *within their submission* to facilitate the interpretation and replication of experiments. If you have any questions, please contact us: [editorial@elifesciences.org](mailto:editorial@elifesciences.org).

**Sample-size estimation**

- You should state whether an appropriate sample size was computed when the study was being designed
- You should state the statistical method of sample size computation and any required assumptions
- If no explicit power analysis was used, you should describe how you decided what sample (replicate) size (number) to use

Please outline where this information can be found within the submission (e.g., page numbers or figure legends), or explain why this information doesn’t apply to your submission:

p. 23 lines 476-84. We sought a testing set showing a superiority of 0.1 in the area under the receiver operating characteristic curve (AUC) against a value of 0.75 (assumed as a null hypothesis for a clinically useful biomarker) with a statistical power of 80% and a type 1 error probability <0.05 (41). For statistical power estimation purposes we assumed that the model predictions would be moderately correlated with CA-125 levels (r>0.3). The calculation yielded a required testing set of 44 patients (22 with invasive cancer and 22 without invasive cancer). To train the classifiers, we assumed the training set would require 3-fold more patients (N=132) bringing the total number of required patient samples to 176 samples. We increased the sample size to 180 to account for potential clinical or technical outliers.

**Replicates**

- You should report how often each experiment was performed
- You should include a definition of biological versus technical replication
- The data obtained should be provided and sufficient information should be provided to indicate the number of independent biological and/or technical replicates
- If you encountered any outliers, you should describe how these were handled
- Criteria for exclusion/inclusion of data should be clearly stated
- High-throughput sequence data should be uploaded before submission, with a private link for reviewers provided (these are available from both GEO and ArrayExpress)

Please outline where this information can be found within the submission (e.g., page numbers or figure legends), or explain why this information doesn’t apply to your submission:

p.20 line 403. miRNA-seq was performed in duplicate for all 179 patient samples.

p.22 lines 450-58 outlines criteria for exclusion/inclusion of data for qPCR

The miRNA-seq raw data are available through the Gene Expression Omnibus (GEO) Accession Series GSE94533.

The calculator described in the manuscript is available at:

<http://biostat.umed.pl/ovaries>

**Statistical reporting**

- Statistical analysis methods should be described and justified
- Raw data should be presented in figures whenever informative to do so (typically when N per group is less than 10)
- For each experiment, you should identify the statistical tests used, exact values of N, definitions of center, methods of multiple test correction, and dispersion and precision measures (e.g., mean, median, SD, SEM, confidence intervals; and, for the major substantive results, a measure of effect size (e.g., Pearson's r, Cohen's d)
- Report exact p-values wherever possible alongside the summary statistics and 95% confidence intervals. These should be reported for all key questions and not only when the p-value is less than 0.05.

Basic statistical analyses: p.32 lines 688-696

Statistical models: p. 24-32

Univariate analyses: Supplementary file 5

Raw, filtered, and normalized data: Supplemental datasets 4-6

Please outline where this information can be found within the submission (e.g., page numbers or figure legends), or explain why this information doesn’t apply to your submission:

(For large datasets, or papers with a very large number of statistical tests, you may upload a single table file with tests, Ns, etc., with reference to page numbers in the manuscript.)

**Additional data files (“source data”)**

- We encourage you to upload relevant additional data files, such as numerical data that are represented as a graph in a figure, or as a summary table
- Where provided, these should be in the most useful format, and they can be uploaded as “Source data” files linked to a main figure or table
- Include model definition files including the full list of parameters used
- Include code used for data analysis (e.g., R, MatLab)
- Avoid stating that data files are “available upon request”

Please indicate the figures or tables for which source data files have been provided:

Sequencing and PCR data are included as supplemental datasets

Computer codes are included as raw pmml files in the supplement
